# Abomasal Nematodes of Goat and In Vitro Evaluation of Anthelmintic Resistance in Arba Minch Zuria District, South Ethiopia

**DOI:** 10.1155/japr/9921528

**Published:** 2025-10-24

**Authors:** Abreham Wondimu, Tamirat Kaba, Yemsrach Wubayehu

**Affiliations:** ^1^College of Agricultural Science, Livestock and Fishery Research Center, Arba Minch University, Arba Minch, Ethiopia; ^2^College of Agricultural Science, Department of Animal Science, Veterinary Program, Arba Minch University, Arba Minch, Ethiopia

**Keywords:** anthelmintic, goat, *Haemonchus*, in vitro, *Teladorsagia*, *Trichostrongylus*

## Abstract

**Aim:**

Goats are a crucial livestock resource for smallholder communities, providing significant livelihood support. However, abomasal nematode parasites in these animals are a major contributor to health problems and productivity losses. This study identified and quantified abomasal nematodes in slaughtered goats, assessed drug usage practices, and evaluated anthelmintic resistance in the Arba Minch Zuria district.

**Methods and Results:**

A cross-sectional and laboratory-based experimental study design was used to collect data from October 2023 to August 2024 through structured questionnaires, postmortem worm recovery techniques, and in vitro drug resistance assays. Accordingly, of 120 examined goat abomasa, 108 (90%) harbored at least one nematode species. *Haemonchus contortus*, *Teladorsagia circumcincta*, and *Trichostrongylus axei* were the worm species identified as sole (21%) or mixed infections (79%). The mean burden of *H. contortus* (757.5) was significantly higher than that of *Teladorsagia circumcincta* (415.8) and *Trichostrongylus axei* (363.3) (*p* < 0.05). Animals' sex, age, and body condition significantly influenced the mean burden of abomasal nematode infection (*p* < 0.001). The survey results reveal the majority of respondents (80%) used albendazole bolus to control parasitism in goats. In vitro assays on *H. contortus* showed that this drug significantly (*p* < 0.05) inhibited egg hatching in a dose-dependent manner, with a maximum rate of 97.9% at 60 *μ*g/*μ*L. The minimum concentration of albendazole that inhibits 50% of egg hatching and embryonation (IC_50_) was 0.0096 *μ*g/*μ*L. Additionally, there was a statistically significant (*p* < 0.05) dose-dependent inhibition of *H. contortus* larval motility observed for ivermectin, with a predicted IC_50_ of 0.257 *μ*g/*μ*L. Moreover, the inhibition of larval motility by tetramisole hydrochloride did not significantly vary among different concentrations, with an expected IC_50_ value of 0.00068 *μ*g/*μ*L.

**Conclusion:**

The high burden of *H. contortus* in the abomasum of slaughtered goats indicates its endemic nature in the area. The higher IC_50_ value for ivermectin suggests a risk of ivermectin resistance in the study area. Therefore, caprine haemonchosis, teladorsagiosis, and trichostrongylosis should be considered during clinical procedures, and combining anthelmintic treatments, creating farmer awareness, and rotating anthelmintic drugs can help reduce the risk of ivermectin resistance.

## 1. Introduction

### 1.1. Background

Goats (*Capra hircus*) are vital livestock for resource-poor farmers in developing countries, providing fiber, manure, and crucial cash income [1]. In Ethiopia, there are approximately 52.5 million goats [2]. These prolific animals make significant contributions to the economy through meat, milk, yogurt, and by-products such as skins [3]. Goats require low capital investment and can efficiently utilize limited, low-quality feed, which makes them preferable to larger ruminants [4]. Moreover, their smaller size and reduced maintenance costs [5] enable them to serve as emergency savings, offering immediate cash income and milk for poor communities reliant on goat rearing [6, 7]. The production and utilization of domestic ruminants in Ethiopia are suboptimal due to numerous constraints, including helminth infections, which pose a global health and economic challenge [8, 9].

Gastrointestinal nematodes, *Haemonchus*, *Teladorsagia*, and *Trichostrongylus*, contribute to morbidity and mortality in tropical and subtropical goats [10], with their impact varying based on agroecological conditions and anthelmintic (AH) susceptibility [11–13]. AHs are widely used in Ethiopia to control gastrointestinal parasitism [14], but reports of anthelmintic resistance (AR) and low efficacy are prevalent due to poor drug quality [15–18]. The primary factor contributing to AR is improper use in livestock production [19, 20]. Assessing AH efficacy in farming localities can provide insights into goat production's therapeutic management of gastrointestinal parasitism. Both in vitro and in vivo methods are used, with organizations like WAAVP and FAO highlighting advantages and disadvantages [21, 22].

In vitro protocols such as the egg hatch assay (EHA) and larval/adult motility tests are standard for detecting nematode resistance to benzimidazoles, imidazothiazoles, and macrocyclic lactones, respectively [23–25]. In Ethiopia, AR to these commonly used drug classes is a growing concern in goats. This decline in efficacy arises from frequent, careless treatment and the misuse of smuggled veterinary drugs [26, 27]. Such resistance severely hinders goat health and productivity and causes significant economic losses, particularly in extensive goat farming regions like the study area [28, 29]. A critical gap exists in farmers' knowledge regarding specific parasites, the impacts of infections, and local AR patterns. Therefore, this study was aimed at assessing drug usage practices, identifying abomasal nematodes, quantifying worm populations, and evaluating AR using in vitro techniques.

## 2. Materials and Methods

### 2.1. Study Area

**Figure 1:**
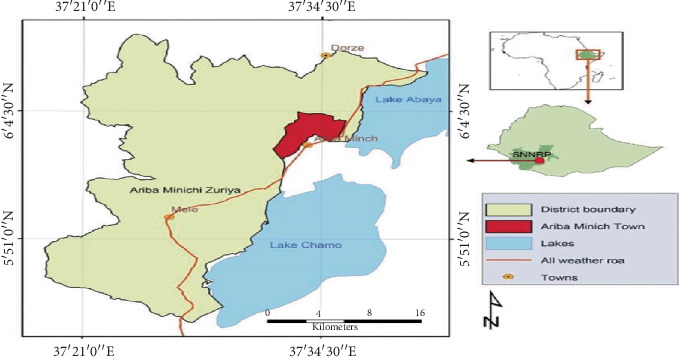
Map of Arba Minch Zuria district, adapted from Abossie et al. [30].

The study was conducted in Arba Minch Zuria district in South Ethiopia (Figure 1). Arba Minch town is the administrative center of the Gamo zone (Figure 1). It is located about 505 km from Addis Ababa, the capital city of Ethiopia. It is located near the center of the region around 5°30⁣^″^–6°50⁣^″^N latitude and 37°32⁣^″^–37°36⁣^″^E longitude cited in [31]. The areas are characterized by a bimodal rainfall pattern with a short rainy season (March to April) and the main rainy season (August to November). The elevation varies from 1108 m to 1285 m.a.s.l. The agroclimatic zone of the study area is characterized by dry Kola (semiarid) and receives 600–1600 mm rainfall per annum; the annual temperature range is 22°C–34°C. The district has a total estimated cattle population of 101,628, a sheep population of 27,339, a goat population of 42,662, a horse population of 3204, and a poultry population of 140,050 reported [32].

### 2.2. Study Animals

The study was conducted on indigenous (*Woito Guji*) breeds of goats from the local market, which were sourced for slaughter in restaurants and butcher shops. Goats of both sexes and various age groups presented for slaughter were enrolled. The age of the animals was estimated by their dentition [33]. For this study, animals under 2 years old were classified as young, while those older than 2 were considered adults. Similarly, the body condition of the goats was categorized as poor, moderate, or good according to the scorecard given by [34].

### 2.3. Study Designs, Sampling, and Sample Size

A cross-sectional and laboratory-based experimental study design was conducted from October 2023 to August 2024 to investigate goat abomasal nematodes and assess AR in vitro. The study comprised three major phases to achieve the objectives of the research.

In Phase 1, *n* = 120 abomasa of goats were collected using nonprobability sampling (convenience sampling) as described by [35] from restaurants and hotels/butcher shops in Arba Minch town and the surrounding area. The number of abomasa required for the study was estimated using Epitools (AusVet) online epidemiological calculator in a single proportion sample size calculation [36], by taking 91.6% expected prevalence of infected abomasa [37], 5% absolute precision, and 95% confidence interval.

In Phase 2, a questionnaire survey was conducted on randomly selected farmers who reside in potential goat farming kebeles of Arba Minch Zuria district to gather data concerning goat husbandry practices, veterinary services, and parasite control measures in the area.

In Phase 3, an in vitro assessment of AR was conducted via experimental design. Young donor goats were purchased from the local market and followed WAAVP guidelines [21, 22]. The AH used in the trial was chosen based on Phase 2 research findings.

### 2.4. Inclusion and Exclusion Criteria

#### 2.4.1. Inclusion Criteria

Those kebeles having different breeds of goats, households, and farmers owning goats were included in the study.

#### 2.4.2. Exclusion Criteria

Households and farmers other than goat owners were excluded from the study.

### 2.5. Abomasum Sampling and Transportation Procedure

Samples were prepared and examined according to Urquhart et al. [38]. Briefly, after slaughtering, the abomasum was removed and separated from other compartments, ligated at omaso-abomasal and pyloric ends, and then transferred directly to the Arba Minch University, Agricultural Science, Veterinary Laboratory, in clean labeled plastic bags/ice boxes.

### 2.6. Worm Recovery, Enumeration, and Preservation

Each abomasum was opened along the greater curvature using a pair of scissors, and the contents were poured into a glass beaker and then processed by repeated washings and sieving until it was clear enough for easier worm counting. The abomasum and the contents were carefully examined, and the contents were then placed in a tray with a label. The contents were gently washed into collecting jars and filtered through a strainer with a 250-*μ*m aperture, capable of retaining the adult worms. The contents were washed into a bucket under running water until the total volume reached 2 L. A 200-mL sample was transferred to a labeled plastic container and preserved in 10% formalin; 20 mL of the subsample was placed on a Petri dish, and the presence of parasites was examined using a stereomicroscope. The number of worms found in 20 mL was multiplied by 100 to calculate the total number of worms found in the abomasum, as described by [39]. The collected adult larvae from the abomasal were preserved in 70% ethyl alcohol for further analysis and identification. It allows the study of the abomasal nematode genus/species in the infected goats and understanding their potential impact on other livestock in the area.

### 2.7. Sample Size for Questionnaire Survey

A structured questionnaire survey was prepared and presented to 100 owners (farmers) having goats interviewed to get information on AH utilization and perceived efficacy. The sample size of the respondents was determined using the formula (*n* = 0.25/SE^2^) proposed by [40] at the standard error (SE) of 0.05 with a 95% confidence interval. The study kebeles were purposively selected based on a relatively large goat population. Afterward, the goat farmers were randomly selected for interview. The questionnaire was designed to obtain information from respondents on drenching practices against GINs, such as husbandry practice, type of AH used, frequency of administration, dosage rate determination, source of AH, criteria for AH selection, and observations on the responses to treatment. Before the in-person interview, the objective of the research was explained to each respondent, and their full consent was obtained.

### 2.8. In Vitro Evaluation of AR

#### 2.8.1. Preparation of Worms

To generate the required number of parasitic stages (eggs, L3) for an in vitro AR assay, adult female worms of the predominant species were collected from the fresh abomasal samples and crushed with a mortar and pestle to liberate the eggs [41]. Before the experiment, the young donor animal was purchased from local markets and acclimatized for 2 weeks. It was then dewormed with an appropriate AH drug to ensure the animal was parasite-free. Again, the donor animal was clinically and parasitologically examined to confirm the absence of infection. Then, eggs were cultured on parasite egg-free animal feces to produce an infective stage of larvae (L3) [42]. After 14 days of culturing at room temperature under sufficient moisture, the larvae were recovered by the modified Baermann technique [43]. After letting the L3 attain its full infective potential for 3 weeks at +4°, they were drenched into the donor animal at the dose rate of 10,000 L3/head per [44]. The animal was handled according to the guidelines for experimental animal use and management [22]. The animals were provided with grass hay supplemented with concentrate (wheat bran). Following the establishment of the infections, fecal collection was done for harvesting L3 to be used in the larval motility test (LMT) and for recovering eggs to be used in the EHA.

#### 2.8.2. AHs Used in This Study

The AHs used for the experiment were bought from local retail markets and composed of three drug classes (Table 1). Before the AHs were purchased, information was gathered on AH utilization practices in the area and the common drugs available on the local market. Then, the efficacy of albendazole (ABZ), ivermectin, and tetramisole [45] was tested using the LMT and EHA methods and interpreted according to the guidelines provided by WAAVP recommendations for efficacy evaluations of AH [21, 22].

#### 2.8.3. EHA

Nematode eggs were isolated from a fresh fecal sample of a donor animal as per the procedures of [46]. Briefly, fecal pellets were collected from the rectum of the donor animal and placed in a small bucket. Warm water was slowly added, and the pellets were stirred until a relatively liquid suspension was obtained. The suspension was strained through a sieve with a 250-*μ*m aperture. The suspension was collected and washed through a 150-pore size sieve once again. The filtrate was then poured into a 15-mL test tube and centrifuged for 2 min at 2000 revolutions per minute (RPM), and the supernatant was decanted. The tube was agitated by a vortex mixer to loosen the sediment. A saturated sugar solution was added to the test tube until the meniscus formed above the tube on which the coverslip was placed. After 5 min, the coverslip was carefully taken off the tube, and the eggs on the surface and edges of the coverslip were washed into glass centrifuge tubes. The tube was filled with water and centrifuged for 2 min at 2000 RPM. The supernatant was siphoned off, and the eggs in the sediment were collected and diluted to the required concentration of drug.

An EHA was done according to the WAAVP guideline [21]. Briefly, locally available AH drugs were diluted in 99.9% DMSO, and a stock solution of each drug was prepared. A 10-fold serial dilution was made to determine the effect of different drug concentrations ranging from 60 to 0.006 *μ*g/*μ*L on egg development. A known 200-*μ*L concentration of egg suspension (roughly 100 eggs) was mixed with various drug concentrations in each 96-well plate. The plate was covered with plastic covering tape and incubated at 27°C for 48 h. After 48 h of incubation, egg development was stopped by adding two drops of Lugol's iodine to each plate to prevent further growth (embryonation). The number of first instar larvae (L1) and unhatched eggs was counted using a stereomicroscope in Petri dishes that have grids for counting. The percentage inhibition of egg hatching was calculated using the formula as follows [47]: %inhibition = (1 − *B*/*A*)∗100, where *A* is the number of larvae and eggs in control and *B* is the number of larvae in different drug concentrations.

#### 2.8.4. Larval Motility Inhibition (LMI) Test

Infectious larvae (L3) were developed following fecal culture obtained from donor animals. It was stored in flat-bottom flasks with a few drops of tap water at room temperature for about 1–2 weeks before the experiment. During the experiment, the L3 was washed three times in deionized water/phosphate-buffered saline (PBS) solution to remove any debris and bacteria/fungi from its body. The concentration of L3 was estimated in the solution and adjusted to the required amount. This test was done to assess the efficacy of macrocyclic lactone (ivermectin) and imidazothiazole (tetramisole) groups [43, 48, 49].

The stock solution of each drug was prepared in DMSO diluent. Different drug concentrations ranged from 60 to 0.006 *μ*g/*μ*L, and controls were prepared through 10-fold serial dilution in a 96-well plate as described above. A 200-*μ*L (roughly 100 L3) clean known concentration of L3 was added to each drug concentration and controlled well. The plate was incubated at room temperature for at least 24 h. Finally, the number of motile and nonmotile L3 [50] was counted, and the % inhibition of larval motility was estimated using the formula %inhibition of larval motility%ILM = (*A* − *B*/*A*∗100), where *A* is the number of motile L3 in the control plate and *B* is the number of motile L3 in each drug concentration. The experiment was conducted in triplicate for each drug concentration.

### 2.9. Data Management and Analysis

The collected data from the parasitological examination was entered into an MS Excel spreadsheet. Descriptive statistics were used to present the questionnaire survey, as well as the prevalence and mean of the abomasum worm feature. Poisson regression in the general linear model was used to assess the statistical association between worm burden and the sex, age, and body condition of the goat. One-way ANOVA followed by Tukey's test was employed to determine statistical significance at *p* < 0.05 for pairwise comparison among several doses. A dose–response curve was created for each in vitro drug efficacy test to get the minimum drug concentration that can kill/inhibit 50% of egg/larva/(IC_50-drug concentration expected to inhibit 50%_) using four-parameter log-logistic dose–response models with “*drc*” package in RStudio (Version 2024.09.1+394). All inferential statistical tests were considered at 5% precision and a 95% confidence level.

### 2.10. Ethical Consideration

In this study, all procedures performed involving animals followed the ethical standards of the institution or practice at which the study was conducted. Participants were informed that they have full rights whether to participate or not in the study. Confidentiality regarding informants' personal information was maintained. A permission letter was obtained from the College of Agricultural Science (Ref. No. AMU/AREC/10/2017) ethical review committee for activities involving goats.

## 3. Results

### 3.1. Control Practices of Gastrointestinal Parasitism in Goats

**Figure 2:**
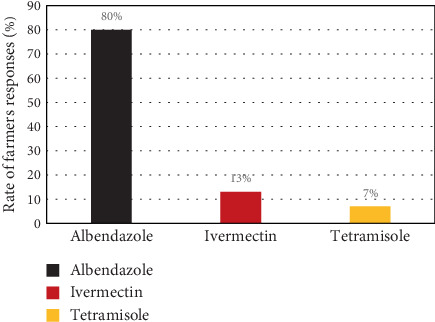
Commonly used anthelmintic drugs in the study area.

The use of AH drugs remains a single available control method of parasitism in goats in the current study area. According to the results, the most commonly used AH drug among the majority of farmers was ABZ, followed by ivermectin and tetramisole (Figure 2). Participants indicated that 52% obtained medications from public veterinary clinics, while 48% sourced them from private clinics or drug vendors (Table 2). Farmers chose AHs primarily based on veterinarians' advice (87%), followed by color (11%) and low price (2%) (Table 2). Almost all farmers (96%) deworm their goats monthly, while the remaining 4% do so twice a year (Table 2).

### 3.2. Abomasal Nematodes in the Slaughtered Goats

**Figure 3:**
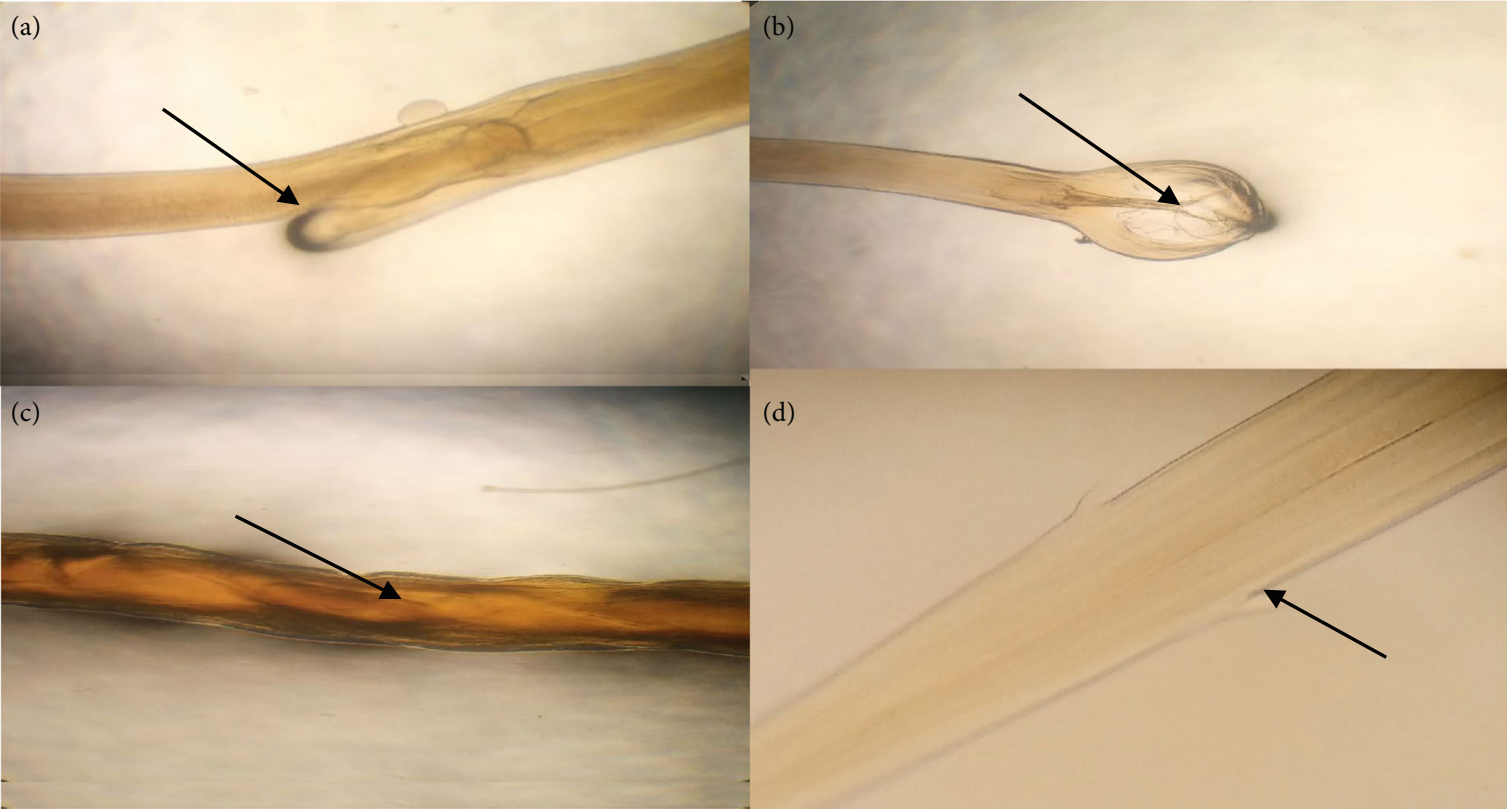
Microscopic identification of *H. contortus* from the abomasum of a goat. Vulvar flap (linguiform) (arrow) of female *H. contortus* (a), spicule (arrow) of male *H. contortus* (b), barber pole appearance (arrow) of female *H. contortus* (c), and cervical papillae (arrow) of *H. contortus* on both sides (d).

**Figure 4:**
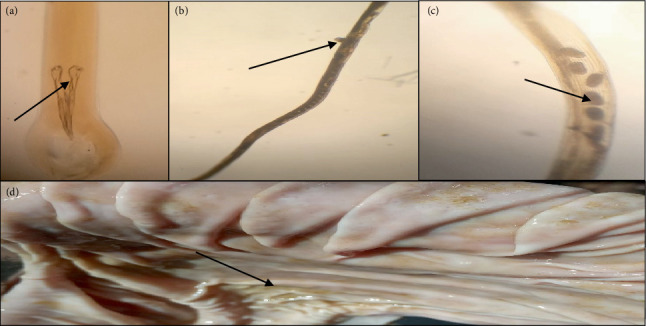
Microscopic identification of *T. circumcincta* from the abomasum of a goat. (a) Spicule (arrow) of *T. circumcincta*. (b) Vulvar flap (arrow) toward the posterior end of the female. (c) Eggs (arrow) toward the posterior end of the female. (d) Lesions (arrow) due to *T. circumcincta*.

**Figure 5:**
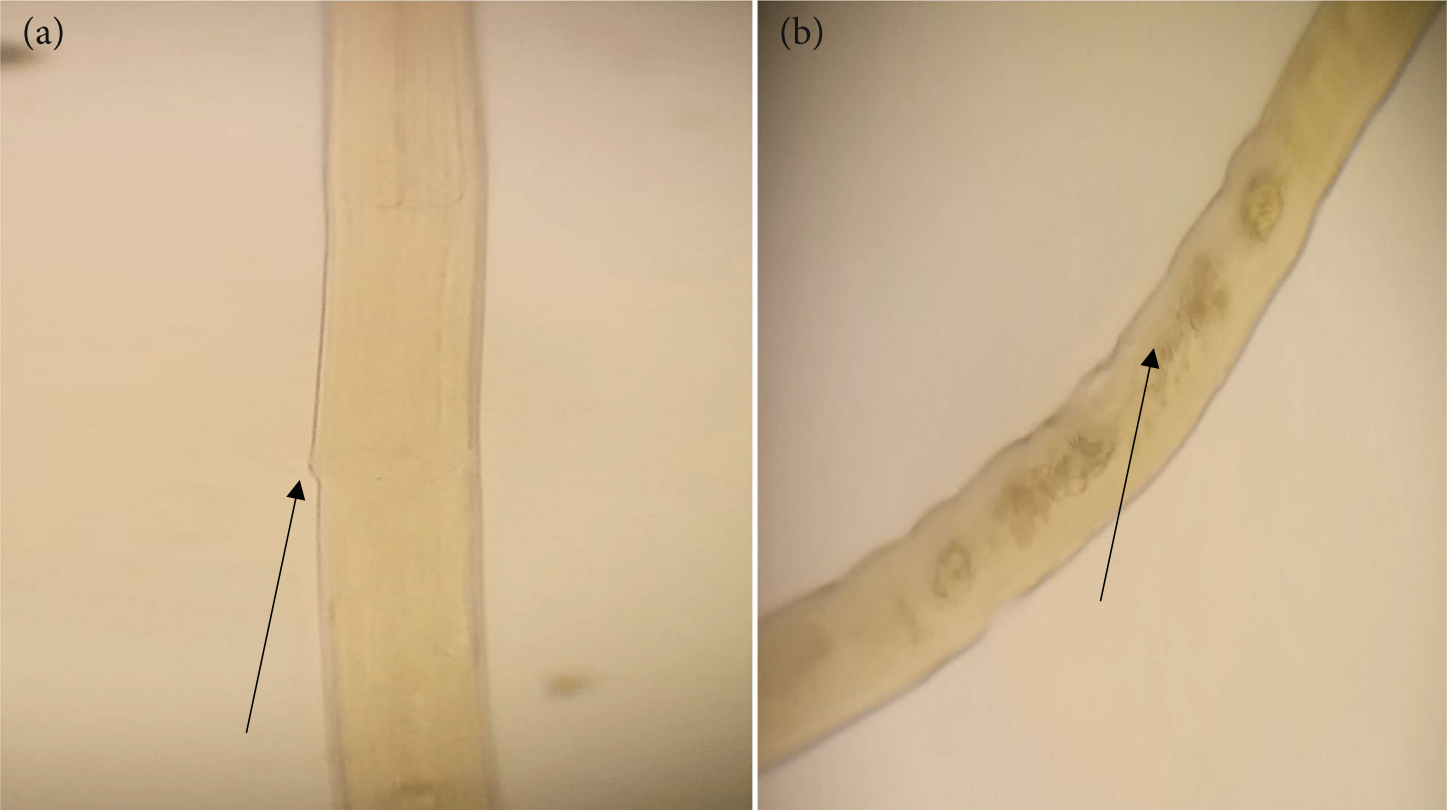
Microscopic morphological identification of *T. axei* from the abomasum of a goat. Excretory notch around the esophageal region (arrow) (a) and ovijector of the female (arrow) (b).

**Figure 6:**
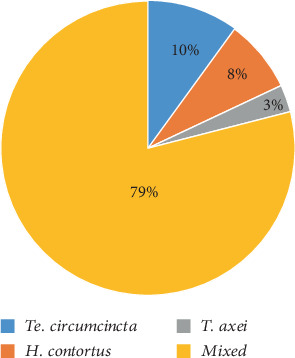
Monospecific and mixed infections of the abomasum of goats with adult nematode worms in Arba Minch Zuria district, Southern Ethiopia.

In a study of 120 examined caprine abomasum samples, 108 (90%) were found to harbor at least one species of nematode. The morphological characterization showed all recovered worms fall into three different species, namely, *Haemonchus contortus*, *Teladorsagia circumcincta*, and *Trichostrongylus axei* (Figures 3, 4, and 5, respectively). A monospecific infection with *H. contortus*, *T. circumcincta*, and *T. axei* accounted for only 21%, whereas 79% of the abomasum had mixed infection with at least two species of nematodes (Figure 6).

### 3.3. Worm Burden

In the present study, *H. contortus* worm was the most abundant parasite with a mean worm burden of 757.5, followed by *T. circumcincta* (415.8) and *T. axei* (363.3). This mean burden was statistically significant (*p* < 0.05) among the parasite population (Figure 4). Considering the sex of the study animals, female animals (790.9) had a higher worm burden than males (750), while young animals (778.11) had higher worm burdens than adults (750) (Table 3). However, concerning body conditioned score, poor (1172.7) conditioned animals had higher worm burdens, followed by medium and good.

### 3.4. Host Factor Association With Overall Worm Count

The Poisson regression model revealed that the host-related variables, such as sex, age, and body condition, significantly (*p* < 0.001) influence the overall worm count ratio in the abomasum of the goat (Table 4). Accordingly, the worm count ratio in females was 1.05 times higher than in males, while it was 1.05 times greater in young compared to adult animals. Regarding body condition, goats with poor body condition had 1.68 times higher worm count than animals with good condition (Table 4).

### 3.5. Evaluation of Common AHs

#### 3.5.1. EHA

**Figure 7:**
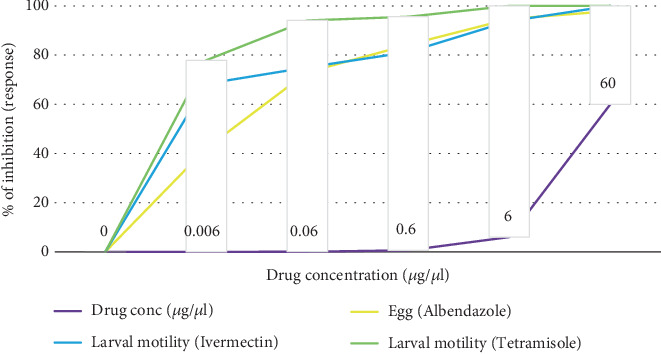
Inhibition (%) of the egg hatching and larval worm motility of *H. contortus* by three anthelmintics.

As shown in Figure 7, there is a significant variation (*p* < 0.05) in the inhibition of hatching of *H. contortus* eggs, which occurs in a dose-dependent manner. The maximum inhibition percentage observed was 97.9% at a concentration of 60 *μ*g/*μ*L of ABZ. A minimum inhibitory concentration that inhibits at least 50% of *H. contortus* egg hatching/embryonation (IC_50_) for ABZ was 0.0096 *μ*g/*μ*L (0.0064–0.0127 *μ*g/*μ*L) (Figure 7).

#### 3.5.2. LMI Assay

Tetramisole showed the ability to inhibit *H. contortus* larval movement, achieving 77.9% inhibition at 0.006 *μ*g/*μ*L and 100% at 60 *μ*g/*μ*L. Although higher concentrations increased inhibition rates, the differences were not statistically significant (Figure 7), suggesting no indication of resistance of the parasite to this drug. In contrast to tetramisole, ivermectin's efficacy was dose-dependent and significant (*p* < 0.05) (Figure 7). The minimum inhibitory concentration that inhibits 50% of larval motility (IC_50_) for tetramisole and ivermectin was 0.00068 *μ*g/*μ*L (95% CI, 0.00035–0.0017 *μ*g/*μ*L) and 0.257 *μ*g/*μ*L (95% CI, 1.14–1.65 *μ*g/*μ*L), respectively (Figure 7). Overall, the results showed that tetramisole outperformed ivermectin in terms of effectiveness against *H. contortu*s larvae.

## 4. Discussions

The use of AH drugs remains the single available control method of parasitism in goats in the current study area. According to the present result, the most commonly used AH drug among the majority of farmers was ABZ, followed by ivermectin and tetramisole. This finding aligns with earlier studies, which indicate that ABZ, an easy-to-administer AH, was preferred over injectable options, as noted by Datiko et al. [51], Seyoum et al. [20], and Negash et al. [52]. Even though farmers in the area of study utilize ABZ as the main AH treatment, tetramisole can also be an important part of a thoughtful goat deworming treatment. This is in contrast to the findings of Alkadir and Ayana [53], who found that ivermectin was the most utilized AH medication, followed by ABZ and tetramisole for treating goats in and around Bishoftu. However, the present study was similar to the result of Dulo and Alaro [54] which reported that farmers in the Humbo district, Wolita zone, utilize ABZ, tetramisole, and ivermectin; this might be due to the widespread availability of the drugs.

Participants indicated that 52% obtained medications from public veterinary clinics, while 48% sourced them from private clinics or drug vendors. This trend may be attributed to the accessibility and lower costs of services, aligning with the findings of Datiko et al. [51] from Bishoftu town. Thirteen percent of farmers self-administer AH drugs without veterinary consultation, indicating a misuse of these medications. Many incorrectly assume that the dosage for goats is the same as for sheep, which aligns with findings by Kamaludeen et al. [55] from New Zealand. This was also consistent with findings from earlier research by Arece et al. [56] who reported from Matanzas, Cuba, that self-administration of AH drugs by a small percentage of goat farmers may stem from practical considerations; it poses significant risks to animal health and overall herd management. Farmers chose AHs primarily based on veterinarians' advice (87%), followed by color (11%) and low price (2%). This approach indicates that they rely heavily on veterinarians for evidence-based recommendations, which can lead to more effective and safer treatment options for parasitic infections. However, many farmers lack the knowledge to accurately estimate their animals' weight, dosage, and injection site.

Almost all farmers (96%) deworm their goats monthly, while the remaining 4% do so twice a year. This consistent use of AH drugs without rotation may contribute to the development of resistance. This finding also aligns with Domke et al. [57], who reported that goat farmers in Northern Italy indicated a low annual number of treatments compared to farmers in other countries, whereas according to Zanzani et al. [58], for farmers from Northern Italy, treatments for gastrointestinal parasites were performed only once a year, which was inconsistent with the present study.

This study examined 120 caprine abomasum samples, finding that 90% (108 samples) harbored at least one nematode species. This prevalence was slightly lower than a previous report from Mekele by Berhe and Aragaw [37] (91.6%), possibly due to environmental changes, but significantly higher than a study in Hawassa [59] (33.1%), which might indicate a lack of deworming strategies or misuse of AHs. Morphological characterization identified three nematode species, namely, *H. contortus*, *T. circumcincta*, and *T. axei*. The presence of *T. circumcincta* differed from a previous Hawassa study of Tesfaheywet and Murga [60], which only found *H. contortus* and *T. axei*, likely due to environmental variations, as these parasites thrive in warm, humid climates [61]. This finding was comparable to an Iranian study of Garedaghi and Bahavarnia [62] (79.5%) that also reported all three species.

A monospecific infection with *H. contortus*, *T. circumcincta*, and *T. axei* accounted for only 21% whereas 79% of abomasum had mixed infection with at least two species of nematodes. Mixed infections were most common; this might be due to a combination of environmental conditions, host behavior, immune response, and nematode life cycle dynamics. The findings of Bersissa et al. [63] and Haileleul [64] from Ethiopia and other countries like Yasser et al. [65], Waruiru et al. [66], and Wang et al. [67] are consistent with this finding. However, this finding was relatively lower and disagrees with previous parasitological findings of Thomas et al. [68] from Hawassa, Argaw et al. [13], from Eastern Hararghe, and Dulo and Alaro [54] from Humbo district, Woliata zone. This might be because the use of strategic AH treatment carried out by trained farmers in the study area was better than the previous.

In the present study, *H. contortus* worm was the most abundant parasite, with a mean worm burden of 757.5, followed by *T. circumcincta* (415.8) and *T. axei* (363.3). This was a similar finding to what Kumsa and Wosene [34] reported in eastern Ethiopia, with the highest mean burden of *H. contortus* from the Ogaden region. This might be due to the similarity of climatic conditions in the study's area. This mean burden was statistically significant (*p* < 0.05) among the parasite population. This was in line with Getachew et al. [69], Kusiluka and Kambarage [70], Maingi et al. [71], and Akhter et al. [72], who reported one possible explanation for *H. contortus*'s dominance among the parasites found: that it was a very prolific parasite, capable of laying thousands of eggs every day for several consecutive months, as long as the environment was favorable and the pasture was highly contaminated compared to the other genera. This finding is also in line with the work of Thomas et al. [68], but it was inconsistent with the finding of Teshale and Aragaw [73], who reported a lower mean worm count (316.5) conducted in Bahir Dar, and Shankute et al. [74], conducted in Debre Zeit (86.5%), central Ethiopia.

Female goat animals had a higher mean worm burden (790.9) than males (750), supporting findings that females are more susceptible to helminth infections due to energy demands during lactation, impairing immunity [75]. Young animals showed higher worm burdens (778.11) compared to adults (750), indicating immature immune responses to nematode parasites. This contrasts with Argaw et al. [12], who found from Eastern Hararghe no significant effect of sex or age on infection rates (*p* > 0.05), suggesting equal susceptibility.

Goats with poor body condition had the highest mean worm burden (1172.7), followed by medium and good condition animals. This aligns with Shankute et al. [74] and other studies [76–79] reporting higher abomasal nematode prevalence and burdens in animals with poor body condition. Heavy parasite infection likely causes weight loss, poor growth, and reduced appetite. A Poisson regression model in animals with poor body condition had 1.68 times higher worm counts than those with good condition. This supports findings that parasite fecundity increases in immunocompromised hosts, while well-nourished animals exhibit stronger immunity that limits parasite burden and reproduction [76–80].

A Poisson regression model revealed that the host-related variables, such as sex and age, significantly (*p* < 0.001) influence the overall worm count ratio in the abomasum of the goat. Accordingly, the worm count ratio in females was 1.05 times higher than in males. This might be due to female animals suffering from stresses during pregnancy and lactation, which may lower their immunity compared to males [81, 82], while it was 1.05 times greater in young animals compared to adult animals. This was in agreement with the work of Gana et al. [75] that adult animals can withstand higher infection without much adverse effect, leading to the chronicity of infection, whereas it coincides with the previous result by Nuruzzaman et al. [83] who reported from Thakurgaon district, Bangladesh, that young animals were more susceptible than the adults. This might be due to the possible explanation that the development of acquired immunity and immune competence increases as age increases, due to the high rate of exposure to parasitic infections. This also supports the idea that young animals are highly susceptible due to immunological immaturity and immunological unresponsiveness [84].

The present study shows that there is a significant variation (*p* < 0.05) in the inhibition of hatching of *H. contortus* eggs, which occurs in a dose-dependent manner. The maximum inhibition percentage observed was 97.9% at a concentration of 60 *μ*g/*μ*L of ABZ. The ovicidal effects of ABZ and the capacity of eggs to grow and hatch at varying drug concentrations served as the basis for this test. The result showed that the eggs failed to develop or hatch with increasing ABZ concentrations. These findings were consistent with those reported by Babjak et al. [85], in Slovakia, and Hafiz et al. [86], in Assam, India. This high level of inhibition could be attributed to the appropriate dosage and handling techniques used, which can enhance effectiveness and help prevent the development of drug resistance.

A minimum inhibitory concentration that inhibits at least 50% of *H. contortus* egg hatching/embryonation (IC_50_) for ABZ was 0.0096 *μ*g/*μ*L (0.0064–0.0127 *μ*g/*μ*L). This result supports the previous finding of Ademola and Eloff [87], who reported from Ibadan, Nigeria, the absence of ABZ resistance in *H. contortus* eggs with the inhibition concentration value of the drug which was less than the cutoff. This means that the effectiveness of ABZ in reducing the number of eggs produced by *H. contortus* varies according to the dosage administered. Higher doses typically lead to a greater reduction in egg counts, while lower doses may be less effective. This is in agreement with the previous results of Mickiewicz et al. [88], who reported from Poland the presence of ABZ resistance (0.78 *μ*g/mL) on eggs laid by *H. contortus.* This might be due to frequent and repeated use of ABZ without rotation or combination with other AHs that can lead to the selection of resistant parasites.

The present study was also consistent with the result of Eguale et al. [89], who reported from Addis Ababa, Ethiopia, the absence of ABZ resistance (0.09 *μ*g/mL) to eggs laid by *H. contortus*. This study generalized that the susceptible *H. contortus* isolate to ABZ, and the hatching and development of eggs decrease significantly with an increase in the concentration of the drug, which was in agreement with the finding of Aboelhadid et al. [90], reported from Egypt. However, this result disagreed with the previous result of Sisay et al. [16], who reported from the eastern region of Ethiopia the presence of resistance to the ABZ drug. This could be because of prolonged usage, improper handling, and underdosing, which are risk factors for both their decreased effectiveness and the growing emergence of drug resistance.

Tetramisole showed the ability to inhibit *H. contortus* larval movement, achieving 77.9% inhibition at 0.006 *μ*g/*μ*L and 100% at 60 *μ*g/*μ*L. Although higher concentrations increased inhibition rates, the differences were not statistically significant, suggesting no indication of resistance of the parasite to this drug. This contrasts with Kumsa et al. [17], who found low efficacy in Southern Oromia. Nevertheless, the results suggest that small doses of tetramisole can effectively impede *H. contortus* movement, potentially minimizing environmental contamination. The relatively higher efficacy of tetramisole among the drugs tested in this study might be due to the low frequency of treatment in the study area. This was in line with the results of Dulo and Alaro [54], who reported no resistance.

Ivermectin showed a 68.8% inhibition of larval motility at the lowest dose. It also achieved 100% inhibition of larval motility in the susceptible strain of *H. contortus* at a concentration of 60 *μ*g/*μ*L. However, it did not induce total inhibition of larvae from parasites isolated from goats, even at the highest concentration tested. This lack of ability to inhibit the motility of *H. contortus* larvae suggests that resistance has developed in the larvae used in this study. According to Coles et al. [21], resistance is indicated by an IC_50_ value greater than 0.1 *μ*g/*μ*L, as per WAAVP guidelines. In contrast to tetramisole, ivermectin's efficacy performance was dose-dependent and significant (*p* < 0.05). This was in contradiction with the finding made by Kamaludeen et al. [55] in New Zealand that *H. contortus* larvae were susceptible. This indicates that the dosage of the medication affects how well these medications kill or inhibit parasitic worms. This was also inconsistent with the Egyptian study of Aboelhadid et al. [90], which found no resistance.

The minimum inhibitory concentration that inhibits 50% of larval motility (IC_50_) for tetramisole and ivermectin was 0.00068 *μ*g/*μ*L (95% CI, 0.00035–0.0017 *μ*g/*μ*L) and 0.257 *μ*g/*μ*L (95% CI 1.14–1.65 *μ*g/*μ*L), respectively. Overall, the results showed that tetramisole outperformed ivermectin in terms of effectiveness against *H. contortu*s larvae. This could be because they have distinct efficacy profiles and modes of action, especially when it comes to certain parasites. This outcome coincides with the earlier finding of Sisay et al. [16], who reported the existence of ivermectin drug resistance in Ethiopia's eastern area. This might be due to the long-term utilization, inappropriate handling, and underdosage, which may be some of the risk factors for their reduced efficacy and the increasing development of drug resistance. A similar situation has been reported by Taylor et al. [10], where parasites have shown resistance to ivermectin at prescribed dosages in goats. Ivermectin does not have any ovicidal activity; however, it does have larvicidal activity according to a report from northeast Punjab by Malik [91]. Nonetheless, there was a low level of AR to ivermectin (2.77 *μ*L/mL) development. This could be due to the continuous use of ivermectin creating selection pressure on helminth populations.

## 5. Conclusions

In conclusion, this study confirmed the widespread presence of abomasal nematodes in goats in the Arba Minch Zuria district, with *H. contortus*, *T. circumcincta*, and *T. axei* as the predominant species, often occurring as mixed infections. Farmers' frequent, yet often irrational, use of AHs such as ABZ, ivermectin, and tetramisole appears to contribute to the development of drug resistance. In vitro evaluations provided strong evidence of potential ivermectin resistance in *H. contortus*, as its IC_50_ surpassed the established threshold. These findings underscore the urgent need for improved AH stewardship, recommending practices such as correct dose administration, drug rotation, strategic treatment timing (especially during the rainy season), and ongoing training for farmers to ensure sustainable parasite control and prevent further resistance development.

## Figures and Tables

**Table 1:** Anthelmintics used in the laboratory efficacy evaluation against goat's nematodes.

**Trade name**	**Generic name**	**Manufacturer**	**Dosage (mg/kg BW)**	**Route**
Albfen-300 mg	Albendazole	Shijiazhuang, China	7.5	Per os
YZTETRA-600 mg	Tetramisole	Hebei Yuanzheng, China	15	Per os
Ivermectin 1%	Ivermectin	Shijiazhuang, China	0.02 ml/kg	Sc^a^

*Note:* Per os (PO): oral route (through the mouth).

^a^Sc: subcutaneous.

**Table 2:** Findings from the survey of respondents.

**Survey questionnaire format**	**Responses**	**Percent**
Source of anthelmintic	Government clinic	52.0
	Private pharmacies	48.0

Reasons for selecting an anthelmintic	Low price	2.0
	Color	11.0
	Veterinary advice	87.0

Frequency of deworming goats	Monthly	96.0
	Twice a year	4.0

Total		100.0

**Table 3:** Average burden (mean) of abomasal worms in the slaughtered goats.

**Variable**	***H. contortus***	***T. axei***	***T. circumcincta***	**Mean total burden**
Sex				
Male	750	352	428.6	1530.6
Female	790.9	413.6	359.1	1563.6
Age				
Young	778.1	390.6	378.1	1546.9
Adult	750	353.4	429.5	1533
BCS				
Poor	1172.7	618.2	590.9	2381.8
Medium	742.3	340.8	376.1	1459.2
Good	685.8	331.6	439.5	1436.8
Overall mean burden	758	416	363	1537

**Table 4:** Association between parasite burden and host-related factors by Poisson regression in a generalized linear model.

**Factors**	**Estimate** ^**a**^ **/count ratio**	**Standard error**	**p** **value**
Sex			
Female	0.053	0.006	0.001
Male	Ref		
Age			
Young	0.05	0.005	0.001
Adult	Ref		
BCS			
Poor	0.522	0.008	0.001
Medium	0.0089	0.005	0.094
Good	Ref		

^a^Estimates are nontransformed log values of Poisson regression in a generalized linear model.

## Data Availability

The authors confirm that all data supporting the findings of this study are included in the article.
